# Induction of Mesenchymal Stem Cell and Preosteoblast Differentiation: A Comparative Study Between Conditioned Medium and In Vitro Coculture

**DOI:** 10.1155/ijod/1123854

**Published:** 2026-06-15

**Authors:** Lina M. Escobar, Zita Bendahan, Wendy Lugo, Eliana Calderón, Luz Eliana Ramirez, Sandra Castaño, Jaime E. Castellanos, Ma Clara Gonzalez-Carrera

**Affiliations:** ^1^ Integral Management Unit of Craniofacial Abnormalities—UMIMC, School of Dentistry, Universidad El Bosque, Bogota, Colombia, uelbosque.edu.co; ^2^ Postgraduate Program in Orthodontics, School of Dentistry, Universidad El Bosque, Bogota, Colombia, uelbosque.edu.co; ^3^ Virology Group, Universidad El Bosque, Bogotá, Colombia, uelbosque.edu.co

**Keywords:** coculture, conditioned medium, differentiation, mesenchymal stem cell, preosteoblast

## Abstract

**Objective:**

To determine and compare the effects on differentiation, proliferation, and morphology of dental pulp‐derived mesenchymal stem cells (DPSC) and preosteoblasts (pre‐OBs) (Saos‐2) that are either cocultured or treated with conditioned medium (CM) in vitro.

**Materials and Methods:**

In this experimental in vitro study, DPSC and Saos‐2 cells were cocultured either directly—allowing physical contact—or indirectly, using a transwell system. These coculture systems were compared to treatment with CM from these cells over varying time periods. Morphological changes were evaluated using light microscopy, while cell proliferation was assessed with the resazurin colorimetric assay. Osteoblastic differentiation was analyzed by Alizarin Red staining and by quantifying changes in Runt‐related transcription factor 2 (RUNX2), osterix (OSX), and osteocalcin (OCN) gene expression via real‐time RT‐PCR. All experiments were performed in triplicate and data analyzed by Student’s *t*‐test, ANOVA, or Mann–Whitney *U* test.

**Results:**

Treatment with CM reduced the cell numbers for both cell types, whereas direct and indirect coculture led to an increase in cell numbers after 8 days of treatment. Morphologically, these treatments induced the formation of more elongated cells with greater cellular extensions compared to control groups. All experimental groups exhibited calcium nodule formation and increased relative expression of RUNX2, OSX, and OCN, consistent with induction of osteogenic differentiation.

**Conclusions:**

The results of this study suggest that both direct and indirect coculture of Saos‐2 and DPSC cells, as well as treatment with CM, are effective in inducing morphological changes, proliferation, and osteoblastic differentiation. These strategies may therefore serve as useful tools for research into regenerative therapies aimed at repairing bone defects.

## 1. Introduction

Bone tissue engineering is an interdisciplinary field focused on developing substitutes to repair or enhance the bone structure and function. Central to this field is the use of stem cells, which possess the unique ability to differentiate into specific tissue types and thereby promote bone formation. This can be achieved either through direct contact between cells and scaffolds or by using conditioned media (CM) obtained from cultured cells. Numerous studies have explored the use of cell cultures to induce bone regeneration by differentiating mesenchymal stem cells (MSCs) into osteogenic lineages, capitalizing on the inherent plasticity of MSCs [1–3].

Within bone tissue, osteoblasts (OBs) reside in close proximity to MSCs, and their combined use has been proposed as a promising strategy for bone repair [4]. The interactions between these cell types can be investigated using coculture systems [5] or by employing CM [6, 7], thereby more closely mimicking the in vivo cellular environment. Although many studies have examined the differentiation of MSCs into OBs and the reciprocal effects of OBs on MSCs [8–10], the influence of MSCs on pre‐OBs and their contribution to bone regeneration remains less well understood, with reports describing both stimulatory and inhibitory effects on osteoblastic differentiation [11–13].

Stem cells secrete a diverse array of bioactive molecules in response to their microenvironment. CM derived from dental pulp‐derived MSCs (DPSC_CM) is the cell‐free culture supernatant collected after a defined period of DPSC culture, which contains the repertoire of bioactive factors released by these cells. Through this complex secreted milieu, DPSC_CM can mediate many of the paracrine and immunomodulatory effects attributed to DPSC, without the need to transplant living cells [14, 15].

These findings may support the development of substitute agents that recapitulate the paracrine effects naturally exerted by cells. Large‐scale production of CM is feasible, offering an abundant source of bioactive molecules without the need for invasive procedures. Moreover, CM‐based therapies present several advantages, including biocompatibility, low tumorigenic potential, reduced risk of thrombus formation, and a minimal risk of infection transmission [16].

Coculture systems, in contrast, involve the cultivation of two or more distinct cell populations together, either in direct contact or separated within a shared environment. This approach is widely used in medicine, cell biology, tissue engineering, pathology, proteomics, and pharmaceutical development. Cocultures enable the study of cellular interactions, infection dynamics, biomimetic environments, cell–cell communication, and stem cell differentiation [17, 18]. These systems can promote tissue formation by supporting stem cell differentiation through the secretion of signaling factors and cytokines by differentiated cells [18, 19].

Two principal types of coculture systems are commonly described: direct and indirect. In direct cocultures, cells share the same environment and engage in cell–cell interactions, which can be assessed in either two‐dimensional or three‐dimensional formats to evaluate adhesion and communication [18]. Indirect cocultures, by contrast, use permeable barriers to physically separate cell populations while allowing the exchange of soluble signaling factors through a shared medium. Although these systems lack direct cell contact, they enable the study of paracrine signaling and have been shown to induce stem cell differentiation via soluble factors [20].

In this study, DPSCs were selected because they are a well‐characterized population of dental‐derived MSCs with robust proliferative capacity, clonogenicity, and proven osteogenic and angiogenic potential, and they have been extensively investigated for craniofacial and alveolar bone regeneration. Their neural crest origin and their native niche inside the tooth make them particularly relevant for endodontic, periapical, and alveolar defect repair, where interaction with OB‐lineage cells at the bone–tooth interface is critical [21]. Additionally, several studies have shown that coculture of DPSCs with OBs or OB‐like cells enhances DPSC proliferation, induces an OB‐like phenotype, and increases mineralization, indicating a strong responsiveness of DPSCs to osteogenic cues provided by bone‐forming cells [22].

Mutual stimulation between MSCs and pre‐OBs is particularly relevant because it models the bidirectional paracrine and matrix‐mediated signals that drive bone formation and remodeling in vivo, and it points toward next‐generation, mechanism‐based regenerative strategies [23]. In coculture, MSCs and OB‐lineage cells exchange cytokines and growth factors that enhance proliferation, migration, and osteogenic differentiation in both populations (e.g., increased alkaline phosphate (ALP), COL1, osteocalcin (OCN)/osteopontin (OPN) expression, and mineralization). This more closely reflects the complexity of the native bone niche than monocultures and is therefore increasingly used to dissect how the osteogenic microenvironment is orchestrated at the cellular and molecular levels. Understanding this crosstalk aligns with current efforts to move from purely cell‐replacement concepts toward microenvironment‐ and signal‐centered therapies in bone regeneration [24].

First, defining the key paracrine mediators in MSC–pre‐OB cocultures (e.g., TGF‐β, VEGF, IL‐6, and specific miRNAs in EVs) can inform acellular approaches using CM or purified EVs, thereby avoiding the need to transplant large numbers of primary cells. Second, these models help optimize preconditioning protocols (inflammatory priming, osteogenic induction, and biomaterial cues) that enhance the pro‐osteogenic secretome of MSCs before clinical application, which has already been shown to promote OB differentiation and mineralization in vitro and bone repair in vivo. Third, insights into mutual stimulation can guide the design of smart biomaterials that fine‐tune MSC–OB signaling at defect sites, improving integration and the long‐term stability of grafts or scaffolds.

Despite these advances, it remains unclear whether CM derived from MSCs or from pre‐OBs is more effective at inducing osteoblastic differentiation in vitro. Likewise, there is insufficient evidence to determine which type of coculture system most efficiently promotes osteoblastic differentiation and bone tissue formation. To dissect the relative contributions of contact‐dependent and paracrine‐only signaling, we employed two complementary coculture configurations. Direct coculture, in which DPSCs and Saos‐2 pre‐OBs share the same surface and establish physical cell–cell contacts, was used to assess the combined effect of juxtacrine signaling (e.g., gap junctions and membrane‐bound ligands) and paracrine factors on osteogenic outcomes. Indirect (transwell) coculture, in which the two cell populations are physically separated by a permeable membrane but share a common medium, was used to isolate the contribution of soluble paracrine mediators in the absence of direct contact. By comparing these setups with CM treatment, we aimed to establish a hierarchy of osteogenic efficacy across the three modes of intercellular communication.

We hypothesized that direct coculture would induce the highest level of osteoblastic differentiation because it integrates both contact‐dependent and paracrine signaling pathways, whereas indirect coculture and CM treatment, which rely solely on soluble factors, would elicit comparatively lower responses. Therefore, the aim of this study was to compare different modes of intercellular interaction—direct coculture, indirect (transwell) coculture, and CM‐based approaches—to identify which strategy most effectively stimulates osteoblastic differentiation in vitro and to clarify the relative importance of contact‐dependent versus paracrine‐only signaling in DPSC–pre‐OB crosstalk.

## 2. Materials and Methods

### 2.1. Isolation of Dental Pulp Stem Cells and Pre‐OBs

This in vitro experimental study employed human DPSCs isolated from premolars extracted for orthodontic indications after obtaining informed consent, together with a human osteosarcoma‐derived preosteoblastic line (Saos‐2; ATCC HTB‐85). This study was approved by the institutional ethics committee under minute number 004‐2022.

DPSC isolation followed the protocol originally described by Gronthos et al. [25], with subsequent modifications as reported by Baldión et al. [26]. Briefly, freshly extracted teeth were surface‐decontaminated by immersion in 5% sodium hypochlorite for 5 s, after which the crowns were sectioned with a high‐speed handpiece to open the pulp chamber and gain access to the radicular pulp. The entire pulp tissue was gently removed and explants were cultured in low‐glucose Dulbecco’s Modified Eagle Medium (DMEM)(HyClone, Thermo Fisher Scientific, Logan, UT, USA) supplemented with 10% fetal bovine serum (FBS) (HyClone, Thermo Fisher Scientific, Logan, UT, USA) and antibiotics. For enzymatic dissociation, pulp tissue was incubated in a digestion solution containing collagenase (3 mg/mL; Sigma–Aldrich, St. Louis, MO, USA) and dispase (4 mg/mL; Gibco, Thermo Fisher Scientific, Paisley, UK) for 16 h at 37°C in a humidified 5% CO_2_ atmosphere. The resulting cell suspension was centrifuged, and the pellet was resuspended in a complete culture medium and seeded into 25 cm^2^ culture flasks until reaching approximately 80% confluence.

DPSCs were initially identified by their adherence to tissue culture plastic and their fibroblast‐like morphology. Immunophenotypic characterization was performed by flow cytometry using 10 µL of fluorochrome‐conjugated monoclonal antibodies directed against CD34, CD45, CD73, CD90, and CD105 (BD Biosciences, San Jose, CA, USA), with unlabeled cells as negative controls, and data acquisition was performed on a BD Accuri C6 cytometer (BD Biosciences, San Jose, CA, USA). A homogeneous mesenchymal population was confirmed by positive expression of CD73, CD90, and CD105 and lack of expression of the hematopoietic markers CD34 and CD45, in accordance with established minimal criteria for MSCs [27].

Functional characterization of DPSCs and Saos‐2 cells was carried out by inducing osteogenic differentiation. Cells were cultured in an osteogenic differentiation medium (ODM), consisting of DMEM supplemented with 0.1 μM dexamethasone, 5 mM β‐glycerophosphate, and 50 μg/mL ascorbic acid, and osteoblastic differentiation was evaluated after 21 days of culture in ODM [28].

### 2.2. Collection of CM

Saos‐2 cells and DPSCs were initially seeded separately in T75 flasks (75 cm^2^ culture area) at a density of 5000 cells/cm^2^ and cultured in DMEM supplemented with 10% FBS until reaching approximately 60% confluence. At this point, cells were rinsed twice with phosphate‐buffered saline (PBS), and the medium was replaced with DMEM containing 10% FBS for the conditioning period. CM was collected every 48 hours over a total conditioning period of 3 days, with 10 mL of medium harvested per flask at each collection. A total volume of 50 mL, obtained by pooling five sequential collections, was prepared for each cell type. To eliminate cellular debris and ensure clarity, the pooled CM was centrifuged at 800 × *g* for 5 min and subsequently filtered through a 0.45 µm membrane filter. The clarified CM was then aliquoted into sterile tubes and stored at −80°C (or at 4°C for short‐term use) until further experimental procedures. It is important to note that the CM was produced under serum‐containing conditions [29]. Therefore, the presence of FBM components indicates that the collected CM represents a mixture of serum‐ and cell‐derived factors rather than a strictly cell‐secreted (serum‐free) CM, which constitutes a limitation in the interpretation of the results. The negative control medium was prepared using fresh culture medium that underwent the same sequence of steps—centrifugation, filtration, aliquoting, and storage—under identical conditions but without any prior exposure to cells, thereby ensuring that potential handling‐ or storage‐related artifacts were equally represented in both preparations.

To ensure batch‐to‐batch consistency and dose equivalence, CM was normalized to the culture area by maintaining a fixed ratio of medium volume to flask surface area (10 mL per 75 cm^2^) and using a standardized seeding density across all conditioning flasks. This approach ensured that each milliliter of CM represented an equivalent secretory output per unit culture area. A fresh medium was replenished every 48 h throughout the collection period.

### 2.3. Direct and Indirect Coculture of DPSCs and Saos‐2 Cells

#### 2.3.1. Direct Coculture

Direct coculture was established by seeding 10,000 DPSCs together with 10,000 Saos‐2 cells (DPSC + Saos‐2) in the same well of 12‐well plates, with experiments performed in triplicate. Cells were maintained in low‐glucose DMEM for 21 days. Negative controls consisted of DPSCs and Saos‐2 cells cultured independently. Positive controls for differentiation included DPSCs or Saos‐2 cells treated with ODM.

#### 2.3.2. Indirect Coculture

For indirect coculture, 12‐well transwell inserts with 0.4 µm pore size PET membranes (Millicell Hanging Cell Culture Inserts, MCHT12H48, Sigma–Aldrich, St. Louis, MO, USA) were used. To assess OB proliferation and differentiation, 10,000 DPSCs were seeded in the bottom wells, while 10,000 Saos‐2 cells were seeded in the inserts (Saos‐2/DPSC). The reciprocal setup, with DPSCs in the insert and Saos‐2 cells in the well (DPSC/Saos‐2), was also performed to maintain a 1:1 cell ratio.

Cell proliferation, viability, and morphology were monitored for up to 8 days of coculture. Osteoblastic differentiation was evaluated at 7, 14, and 21 days. DPSCs and Saos‐2 cells cultured alone served as negative controls, while cells treated with ODM during the same time points served as positive controls.

### 2.4. Determination of Proliferation Rate of DPSCs and Saos‐2 Pre‐OBs

Cell proliferation and viability were quantified using the trypan blue exclusion assay, counting viable and nonviable cells with a hemocytometer. In parallel, a resazurin‐based fluorometric assay was performed by incubating cultures with resazurin (0.44 μM per well) at 37°C for 4 h, followed by fluorescence measurement on a Tecan Infinite M2000 Pro plate reader at excitation/emission wavelengths of 535/595 nm [30].

### 2.5. Evaluation of Osteoblastic Differentiation of DPSCs and Saos‐2 Induced by CM or Coculture

#### 2.5.1. Assessment of Calcium Deposition and Matrix Mineralization

Calcium deposition and extracellular matrix mineralization were assessed according to the Alizarin Red S staining method described by Gregory et al. [31]. After 7, 14, and 21 days of direct or indirect coculture, or after treatment with CM, cells were fixed with paraformaldehyde and stained with 2% Alizarin Red S (Sigma–Aldrich, St. Louis, MO, USA). Excess dye was removed with PBS washes, mineralized nodule formation was visualized by inverted light microscopy, and the bound dye was quantified spectrophotometrically at 550 nm using a Tecan Infinite 200PRO reader.

The Alizarin Red absorbance value obtained for each experimental group was normalized to the cell number determined from matched parallel wells.

#### 2.5.2. Gene Expression Analysis of Osteogenic Markers

Total RNA was isolated from DPSCs and Saos‐2 cells after 7, 14, and 21 days of coculture or CM treatment using the Quick‐RNA MicroPrep kit (Zymo Research, Irvine, CA, USA), following the manufacturer’s protocol.

In the direct coculture, RNA was isolated from a mixed population of DPSCs and Saos‐2, and the results are interpreted as the effect of the simultaneous growth of both cell populations (DPSC + Saos‐2).

First‐strand complementary DNA (cDNA) was synthesized with the ProtoScript II First Strand cDNA Synthesis kit (New England Biolabs, Ipswich, MA, USA). Quantitative real‐time PCR (qRT‐PCR) was carried out to determine the expression of osteogenic markers Runt‐related transcription factor 2 (RUNX2), OCN, and osterix (OSX) using the Luna Universal qPCR kit (New England Biolabs, Ipswich, MA, USA) and a CFX96 Real‐Time PCR system (Bio‐Rad, Hercules, CA, USA). The cycling program consisted of an initial denaturation at 95°C for 3 min, followed by 50 cycles of 95°C for 10 s, 60°C for 30 s, and 72°C for 20 s, and a melt curve step from 65°C for 5 s to 95°C for 5 s. Primer sequences are presented in Table 1. PCR efficiency was calculated with LinRegPCR software (Academic Medical Center, Amsterdam), and relative gene expression was determined using the method described by Schefe et al. [32]. Fold change was calculated using a formula that incorporates amplification efficiency, yielding more accurate values than the conventional ΔΔCt approach. Glyceraldehyde 3‐phosphate dehydrogenase (GAPDH) was used as the reference gene and remained stable across all experimental conditions and time points, indicating its suitability for normalization in these cells. Changes observed over different days were obtained relative to the control group, consisting of unstimulated cells. The reported values represent the mean of three replicates per condition at each time point.

**Table 1:** Sequence of primers used.

Gen	Forward	Reverse
RUNX2	CATCTAATGACACCACCAGGC	GCCTACAAAGGTGGGTTTGA
OSX	TGGGAAAAGGGAGGGTAATC	CGGGACTCAACAACTCTGG
OCN	CCTCACACTCCTCGCCCTAT	TCCCAGCCATTGATACAGGT
GAPDH	GAAGGTGAAGGTCGGAGTC	GAAGATGGTGATGGGATTTC

Abbreviations: GAPDH, glyceraldehyde 3‐phosphate dehydrogenase; OCN, Osteocalcin; OSX, Osterix; RUNX2, runt‐related transcription factor 2.

### 2.6. Statistical Analysis

Statistical analyses were conducted using IBM SPSS Statistics version 27 (IBM SPSS, Chicago, IL, USA). For each outcome variable, descriptive statistics (mean and standard deviation [SD]) were calculated across experimental groups. Data distribution was evaluated with the Shapiro–Wilk test and homogeneity of variances with Levene’s test. When parametric assumptions were satisfied, group comparisons were performed using one‐way ANOVA, followed by Tukey’s honestly significant difference (HSD) post hoc test. In cases where normality or homoscedasticity criteria were not met, nonparametric comparisons were carried out using the Kruskal–Wallis test with the Dunn’s procedure for multiple comparisons. Results are expressed as mean ± SD, and differences were considered statistically significant at *p* ≤ 0.05. All experiments were performed using two independent cell cultures (biological replicates), each with three measurements within the same culture (technical replicates) per experimental condition, and results are reported as the mean ± SD.

## 3. Results

### 3.1. Characterization of MSCs Isolated From Dental Pulp

**Figure 1:**
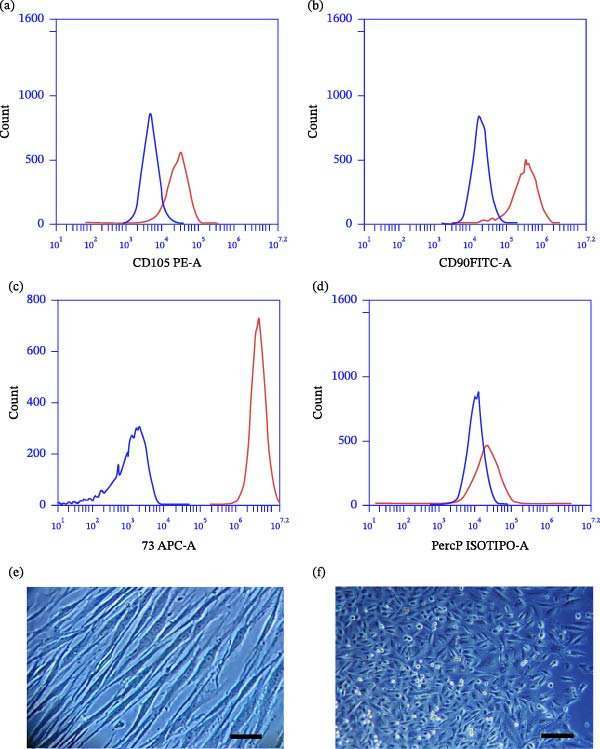
Isolation and characterization of dental pulp stem cells (DPSCs): flow cytometry histograms showing positive expression of CD105 (a), CD90 (b), and CD73 (c), and negative expression of CD34 and CD45 (d). Photomicrograph of DPSCs with fibroblast‐like morphology (e) and Saos‐2 cells (f). Scale bar = 200 µm.

MSCs were successfully obtained from human dental pulp tissue. Flow cytometric immunophenotyping showed robust expression of the mesenchymal markers CD105 (Figure 1a), CD90 (Figure 1b), and CD73 (Figure 1c), with the absence of the hematopoietic antigens CD34 and CD45 (Figure 1d). Morphologically, the cells exhibited a spindle‐shaped, fibroblast‐like phenotype and displayed strong adhesion to the tissue culture surface (Figure 1e).

By contrast, Saos‐2 cells, an established osteosarcoma cell line derived from the primary tumor of an 11‐year‐old Caucasian girl in 1973 (ATCC HTB‐85), were used as received and were not subjected to additional functional or immunophenotypic characterization within this study (Figure 1f).

### 3.2. Effects on Cell Number and Proliferation in Saos‐2 and DPSCs Under Coculture and CM Treatments

**Figure 2:**
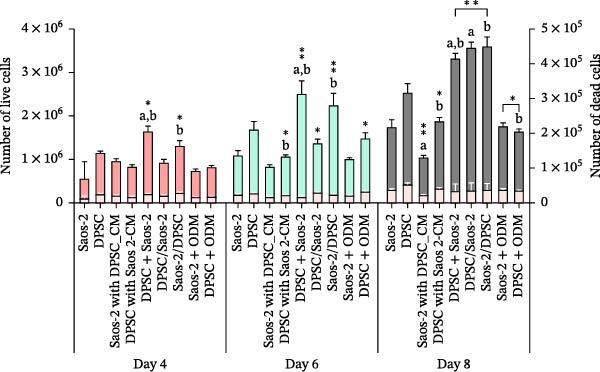
Effect on cell number induced by conditioned medium treatment (CM), direct and indirect coculture. Cell viability and proliferation analysis was performed for 8 days and data were expressed as averages +/−SD. Statistical significance was determined for each time and experimental group relative to its negative control (untreated cells): “a” represents Saos‐2 and “b” represents DPSC. Pink bars represent the number of dead cells in each experimental group. Asterisks represent significant differences with other experimental groups evaluamted,  ^∗^ *p*  < 0.05 and  ^∗∗^ *p*  < 0.01. Direct coculture (DPSC + Saos‐2) and indirect coculture (DPSC/Saos‐2 and Saos‐2/DPSC), osteogenic differentiation medium (ODM).

Treatment of Saos‐2 cells with DPSC_CM, and treatment of DPSCs with CM from Saos‐2 cells (Saos‐2‐CM) resulted in a significant reduction in cell number. This decrease was first observed at 6 days in DPSCs cultured with Saos‐2‐CM (*p*  < 0.05), with a significant reduction in proliferation evident by day 8 in both CM‐treated cell types (*p*  < 0.05 for DPSCs treated with Saos‐2‐CM and *p*  < 0.01 for Saos‐2 cells treated with DPSC_CM). Importantly, no significant cell death was detected (less than 5%), indicating that the reduced cell number was due to decreased proliferation rather than cytotoxicity (Figure 2). This pattern is consistent with a differentiation‐associated slowdown of cell cycle progression, which may occur as cells commit to an osteogenic lineage under CM treatment and ODM stimulation.

Conversely, both direct coculture (DPSC + Saos‐2) and indirect coculture conditions (DPSC/Saos‐2 and Saos‐2/DPSC) promoted a significant increase in cell proliferation from 4 days post‐treatment. Notably, an increase in cell number was observed as early as day 4 in the direct coculture group (DPSC + Saos‐2) and the indirect coculture group where Saos‐2 cells were cultured with DPSCs (Saos‐2/DPSC) (Figure 2).

### 3.3. Morphological Changes in Saos‐2 and DPSC Cells Following CM Treatment and Coculture

Cells treated with CM exhibited a more elongated, fibroblast‐like morphology and lower cell density compared to those of control groups. In direct coculture (Saos‐2 + DPSC), an increase in cell density was observed, with DPSCs displaying a predominant fibroblast‐like phenotype and prominent cytoplasmic extensions.

In the indirect coculture (DPSC/Saos‐2), Saos‐2 cells exhibited morphological changes characterized by a more elongated shape and less triangular appearance compared to controls. Conversely, in the Saos‐2/DPSC coculture, both an increased cell number and more pronounced cytoplasmic extensions were observed.

**Figure 3:**
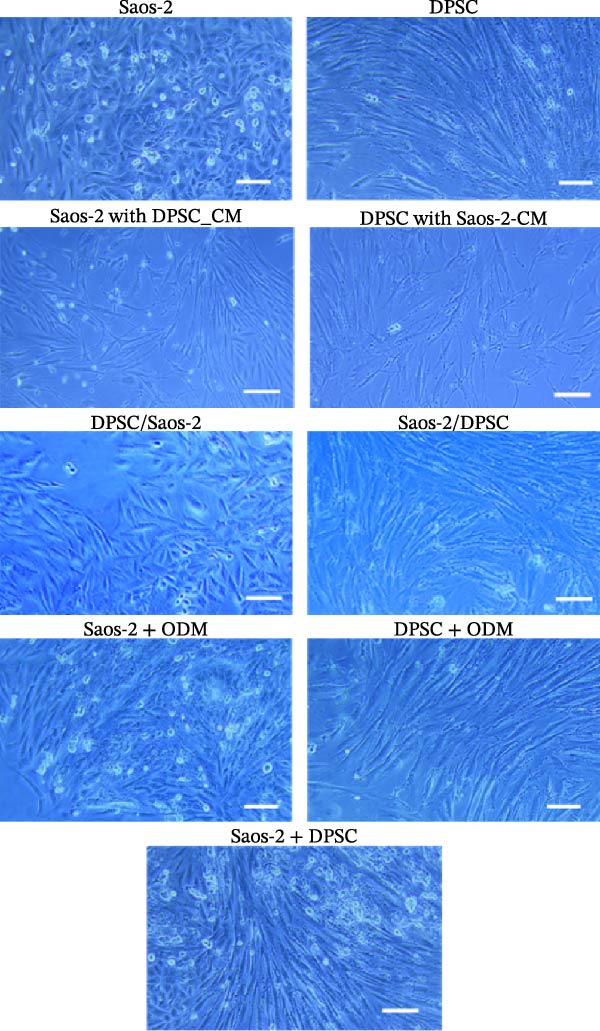
Photomicrograph of Saos‐2 and DPSC cell cultures. Morphological changes induced by direct coculture (Saos‐2 + DPSC) and indirect coculture (DPSC/Saos‐2 and Saos‐2/DPSC) were evaluated. In addition, morphological alterations were assessed in Saos‐2 cells treated with DPSC‐derived conditioned medium (DPSC_CM) and in DPSC treated with Saos‐2‐derived conditioned medium (Saos‐2‐CM). Images correspond to cells after 8 days of culture. Osteogenic differentiation medium (ODM). Bar 200 μm.

The morphological phenotype of cells treated with CM or cultured in direct or indirect coculture conditions resembled that of cells treated with ODM, characterized by increased cytoplasmic prolongations and a fibroblast‐like appearance (Figure 3).

### 3.4. Evaluation of Osteogenic Differentiation Stimulated by CM Treatment and Direct or Indirect Coculture of Saos‐2 and DPSC Cells

Alizarin Red S staining was performed to assess the formation of calcified nodules, indicative of osteogenic differentiation. Microscopic analysis revealed strong Alizarin Red S staining in DPSCs and Saos‐2 cells treated with CM, as well as in direct cocultures (Saos‐2 + DPSC) and indirect cocultures (DPSC/Saos‐2 and Saos‐2/DPSC). The intensity of staining was directly proportional to the duration of treatment for each cell type. In contrast, untreated (control) DPSCs and Saos‐2 cells exhibited no Alizarin Red S staining. The presence of Alizarin Red S staining indicates matrix mineralization and calcium nodule formation, hallmarks of osteoblastic differentiation.

**Figure 4:**
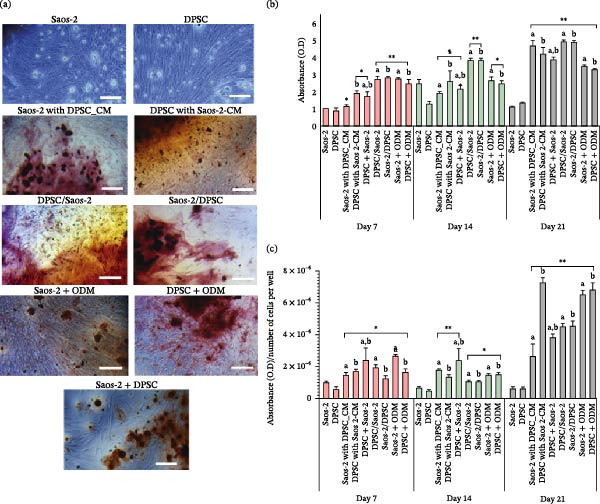
Mineralization of the extracellular matrix. (a) Mineralization was determined by Alizarin Red staining, with strong staining of the matrix in the microphotographs indicating the apparent formation of calcification nodules. (b) Raw values of absorbance of Alizarin Red staining extracted from conditioned medium‐treated cells and direct (DPSC + Saos‐2) and indirect (DPSC/Saos‐2 and Saos‐2/ DPSC) coculture at 7, 14 and 21 days. (c) Normalized Alizarin Red absorbance value per cell number in each well. Untreated cells (control), osteogenic differentiation medium (ODM). The data are expressed as averages +/−SD. Statistical significance was determined for each time and experimental group relative to its negative control (untreated cells): “a” represents Saos‐2 and “b” represents DPSC. Asterisks represent significant differences with other experimental groups evaluated,  ^∗^ *p*  < 0.05 and  ^∗∗^ *p*  < 0.01.

A comparison of the experimental groups revealed that indirect coculture (Saos‐2/DPSC and DPSC/Saos‐2) resulted in the highest formation of calcium nodules, starting at 14 days of treatment. In all experimental groups, calcified nodule formation was evident after 7 days of treatment, relative to the control group (Figure 4a).

Quantification of Alizarin Red S staining, following dye solubilization and extraction, demonstrated that the highest absorbance (O.D) values, indicative of increased calcium deposition, were observed in both DPSCs and Saos‐2 cells at 21 days of treatment with CM and indirect coculture (DPSC/Saos‐2 and Saos‐2/DPSC). Values obtained with ODM and direct coculture (Saos‐2 + DPSC) were statistically significant after 7 days compared to the control, but absorbance values were lower than those obtained with CM and indirect coculture (Figure 4b). Subsequently, the Alizarin Red absorbance values were normalized to the number of cells per well, revealing significant differences from day 7 onward in all experimental groups compared with the control groups (DPSC and Saos‐2) (Figure 4c).

**Figure 5:**
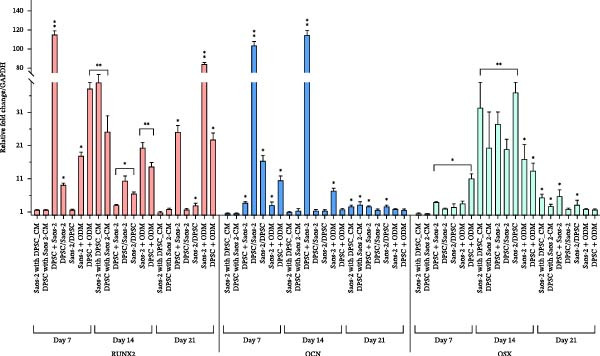
Quantification of the relative expression of RUNX2, OSX, and OCN genes. Quantification of RUNX2, OSX, and OCN expression was performed in cells treated with conditioned medium (CM), direct coculture (DPSC + Saos‐2), and indirect coculture (DPSC/Saos‐2 and Saos‐2/DPSC) for 7, 14, and 21 days. Data are expressed in relation to GAPDH gene expression levels and cells treated with osteogenic differentiation medium (ODM) were analyzed as a positive differentiation control.  ^∗^ *p*  < 0.05 and  ^∗∗^ *p*  < 0.01.

Additionally, changes in the expression of differentiation markers RUNX2, OSX, and OCN were determined using real‐time qPCR analysis (Figure 5).

Saos‐2 cells treated with DPSC_CM exhibited a marked increase in RUNX2 expression, with a 46‐fold elevation at 14 days and a 6‐fold increase in OCN expression at the same time point. In DPSCs treated with Saos‐2‐CM, RUNX2 expression was elevated starting at 7 days, reaching a significant 20‐fold increase at 14 days, followed by a decline at 21 days. OCN expression in these DPSCs showed a modest increase at 7 days, doubled at 14 days, and decreased by 21 days.

In direct coculture (DPSC + Saos‐2), RUNX2 and OCN transcript levels measured in the combined cell population showed a robust increase as early as 7 days, with RUNX2 reaching a 116‐fold elevation and OCN peaking at 14 days with a 111‐fold elevation relative to controls. In indirect coculture conditions (DPSC/Saos‐2 and Saos‐2/DPSC), both RUNX2 and OCN expression increased at 7 days. By 14 days, OCN expression remained elevated, while RUNX2 expression declined. At 21 days, the expression levels of both genes decreased in these groups. The treatment of both DPSCs and Saos‐2 cells with ODM resulted in sustained RUNX2 upregulation from 7 to 21 days. In contrast, OCN expression remained elevated until 14 days and decreased by 21 days. On the other hand, OSX expression began to increase at 7 days in both the direct and indirect coculture groups as well as in cells treated with ODM. Its increase was more pronounced at 14 days in all groups, whereas the OSX expression decreased by 21 days of treatment (Figure 5).

## 4. Discussion

MSCs have emerged as promising therapeutic agents due to their multipotent differentiation capacity, direct regenerative effects in tissues such as bone, and potent wound‐healing abilities mediated by their immunosuppressive and anti‐inflammatory properties. It is well established that MSCs secrete a complex secretome, which may facilitate the transfer of regulatory molecules essential for paracrine and endocrine induction of tissue repair [33].

While numerous studies have investigated MSC differentiation and the influence of OBs on MSCs [8–10], the reciprocal effects of MSCs on OBs and their impact on bone regeneration remain less explored and somewhat controversial. Coculture systems have been employed to study these interactions as OBs and osteocytes are known to produce paracrine factors that promote osteoblastic differentiation of MSCs [9]. However, a direct comparison of the efficiency of CM treatment versus coculture in inducing MSC or pre‐OB differentiation in vitro has not been thoroughly investigated.

In this study, we evaluated the effects of CM treatment, as well as direct and indirect coculture, on the proliferation, viability, morphology, and differentiation of DPSCs and Saos‐2 pre‐OB cells in vitro. Morphological analysis revealed that Saos‐2 cells treated with DPSC_CM became more elongated, spindle‐shaped, and larger compared to those of controls. After 8 days of CM treatment, Saos‐2 cells lost their typical triangular morphology and exhibited a fibroblastoid phenotype. Similarly, DPSCs cultured with Saos‐2‐CM displayed an increased size, extended cytoplasmic processes, and a fibroblast‐like appearance. These morphological changes are consistent with those induced by known osteogenic differentiation agents, such as ascorbic acid [30].

In indirect coculture (DPSC/Saos‐2), Saos‐2 cells adopted a more elongated and less triangular morphology. Conversely, in the Saos‐2/DPSC indirect coculture, an increase in the cell number and cytoplasmic prolongations was observed. The phenotypes observed under CM treatment and both coculture conditions closely resembled those induced by ODM, which served as a positive control. Wang et al. [22] reported that osteogenic differentiation induced by OB and DPSC in indirect coculture resulted in morphological changes from fusiform to elongated shapes beginning at day 7 [34]; our study observed such changes as early as day 4 in both direct and indirect cocultures.

Regarding proliferation, treatment with DPSC_CM reduced Saos‐2 cell numbers at 8 days, while DPSCs treated with Saos‐2‐CM showed significantly reduced proliferation between days 6 and 8 compared to that of controls. ODM treatment also reduced cell numbers starting at day 6. These findings differ from those of Kunimatsu et al. [34], who observed increased proliferation in human bone marrow MSCs (hBMSCs) and mouse calvarial osteoblastic cells after 48 h of treatment with CM derived from stem cells from human exfoliated deciduous teeth pulp (SHED‐CM) MSCs. Similarly, Vu et al. [35] reported enhanced proliferation, survival, and migration of DPSCs treated with SHED‐CM in a dose‐dependent manner, along with upregulation of osteogenic markers, including ALP, OCN, and RUNX2 after 48 h.

In coculture experiments, Wang et al. [22] observed an increased proliferation of DPSCs cocultured with OBs. Consistent with this, our study demonstrated that cocultured cells exhibited significantly increased proliferation, with direct coculture effects evident by day 4 and indirect coculture effects by day 6. Notably, in the CM groups, we observed an apparent pattern of reduced proliferation concomitant with higher osteogenic readouts. This profile is compatible with the well‐described slowdown in cell growth that accompanies osteogenic commitment and progression toward terminal differentiation rather than necessarily indicating cytotoxicity or loss of cell fitness. To minimize this potential confounding factor, all osteogenic readouts were normalized to cell number at each time point so that increases in osteogenic markers and mineralized matrix primarily reflect differentiation‐associated changes rather than simple differences in cell density.

When evaluating calcium nodule formation using Alizarin Red staining, we observed a significant increase in these nodules primarily after 21 days of CM treatment in both cell types. These findings are consistent with those reported by Kim et al. [36], who studied the effects of DPSC_CM on the proliferation and differentiation of human dental pulp stem cells (HDPCs) and OBs (MG63). Their study demonstrated a significant increase in calcium nodule formation in both HDPC and MG63 cells treated with the secretome for 21 days. Although the ODM also induced nodule formation, it was less effective than the HDPC secretome [36]. Similarly, our results show pronounced calcium nodule formation stimulated by CM treatment in both DPSC and Saos‐2 cells, exceeding that observed in ODM‐treated cells.

Osteogenic differentiation was further assessed by measuring the expression of marker genes RUNX2, OSX, and OCN. Both Saos‐2 cells and DPSCs treated with CM showed a significant increase in RUNX2 and OCN expression after 14 days. In direct coculture, the combined RUNX2 and OCN expression profiles of the mixed DPSC + Saos‐2 population progressively increased from day 7 to day 21. In indirect cocultures (DPSC/Saos‐2 and Saos‐2/DPSC), both genes showed elevated expression on day 7; thereafter, OCN remained high, while RUNX2 declined by day 14. ODM treatment caused a sustained increase in RUNX2 expression from day 7 to day 21, whereas OCN levels stayed elevated until day 14 but decreased by day 21. OSX expression increased from day 7 in both direct and indirect coculture groups, and this increase became more evident at 14 days of treatment in all experimental groups. These patterns align with Kunimatsu et al. [34], who reported that SHED‐CM enhanced ALP, RUNX2, and OCN expression in MC3T3 osteoblastic cells compared to cells cultured with 10% FBS. However, SHED‐CM did not significantly affect these markers in hBMSCs after 48 h [34].

Our study also found a significant increase in OCN expression, paralleling Kim et al. [36], who observed elevated OCN from day 15 to day 25 in HDPC and MG63 cells treated with HDPC‐CM. Notably, this treatment promoted HDPC proliferation but reduced MG63 proliferation. They also reported a significant rise in ALP activity after 7 days, indicating early osteogenic differentiation, consistent with our observations in DPSC and Saos‐2 cells [36].

While the bone regeneration effects of DPSC_CM remain underexplored, most research has focused on CM from SHED [34] and stem cells from the apical papilla (SCAPs) [37]. SHED‐CM accelerates cell growth and increases the mRNA expression of osteogenic markers ALP, OCN, and RUNX2 in hBMSCs and MC3T3‐E1 cells in vitro. Chouaib et al. [38] demonstrated that DPSC_CM enhances proliferation, upregulates osteogenic genes (RUNX2, ALP, andOCN), and promotes calcium nodule formation, findings that closely mirror ours.

The paracrine effects of MSCs and preosteoblastic cells on OB differentiation during bone regeneration are not well understood. In this study, we used a transwell system to investigate the effects of DPSCs on OB‐like cells (Saos‐2) and vise versa. This system permits the diffusion of soluble factors between separated compartments while preventing direct cell‐to‐cell contact, thereby isolating paracrine effects from direct interactions. Using this approach, we observed increased proliferation in both cell types, enhanced calcium nodule formation, and elevated RUNX2, OSX, and OCN gene expression. These results demonstrate that indirect coculture efficiently stimulates osteoblastic differentiation in both DPSCs and Saos‐2 pre‐OBs. Comparable findings were reported by Chen et al. [39], who showed that periodontal ligament stem cells (PDLSCs) increased ALP activity, expression of ALP, bone sialoprotein (BSP), OPN, and mineralized matrix deposition in MC3T3‐E1 pre‐OBs using a noncontact coculture system.

In the study by Kim et al. [40], potential synergistic interactions in osteogenesis were evaluated between two cell types: rabbit bone marrow‐derived MSCs cocultured with rat calvarial OBs in direct cell‐to‐cell contact under both control and osteogenic media conditions. The authors reported that cell number and ALP activity in the coculture were intermediate between those observed in the OB and MSC monocultures. However, calcium deposition in the coculture was lower than in the individual cultures [40]. In contrast, our results demonstrate that coculturing mesenchymal cells with pre‐OBs induces calcium nodule formation comparable to that seen in positive controls treated with an ODM. Additionally, we observed a significant upregulation of differentiation marker genes such as RUNX2 and OCN. These discrepancies may stem from differences in cell types used as previous reports suggest that mature OBs may be less effective osteogenic inducers than pre‐OBs [40].

Similarly, Birmingham et al. [9] showed that OBs strongly promote MSC proliferation during the early culture phases, followed by increased ALP activity and osteogenic differentiation. Their three‐layer coculture system revealed a synergistic relationship between osteocytes and OBs, producing biochemical signals that stimulate MSC osteogenesis. This provided valuable insights into the native stem cell niche mechanisms that regulate osteogenic differentiation [9]. Wang et al. [22] further demonstrated that mature OBs significantly influence MSC differentiation at both transcriptional and protein expression levels. In MSCs cocultured with OBs, expression of osteoblastic differentiation genes—RUNX2, OSX, and BSP—was elevated, and calcium deposition increased by 1.6‐fold compared to MSC monocultures [22].

Bian et al. [41] compared osteogenesis and angiogenesis in transwell (indirect) versus direct coculture systems using human amniotic membrane‐derived MSCs (HAMSCs) and hBMSCs. They found that cell proliferation, osteogenesis, and angiogenesis were enhanced in the indirect coculture system relative to direct coculture. Moreover, phosphorylation of the ERK1/2 MAPK signaling pathway—a key regulator of these processes—was greater in the indirect coculture [41]. In our study, increased cell proliferation was observed in both coculture types; however, this increase occurred earlier (at 4 days) in the direct coculture of dental pulp stem cells (DPSCs) with Saos‐2 cells. Both culture systems induced calcified nodule formation, although Alizarin Red staining was more intense in the transwell (indirect) coculture than in direct coculture or ODM‐treated cells. Osteogenic differentiation in both systems was accompanied by a significant upregulation of RUNX2 and OCN, particularly at 7 and 14 days post‐treatment.

Collectively, these findings suggest that treatment with CM, as well as direct and indirect coculture of Saos‐2 cells with DPSCs, are effective strategies to promote osteoblastic differentiation, with potential applications in regenerative therapies for bone repair. The choice of the method should consider its respective advantages and limitations. CM contains soluble factors that can stimulate osteogenesis without requiring direct cell contact, making it useful for enhancing the osteoinductive properties of biomaterials. However, the composition and concentration of these factors can vary, potentially leading to inconsistent effects compared to direct cell interactions [42]. Direct coculture enables cell‐to‐cell contact, which can amplify osteogenic differentiation through synergistic interactions between different cell types, such as OBs and MSCs. This approach often results in more robust osteogenesis due to combined soluble and contact‐mediated signaling; however, the efficiency may be influenced by the ratio of cell types and the presence of osteogenic supplements. Notably, a higher density of certain cells can inhibit osteogenesis [10, 43]. Indirect coculture systems, where cells share a medium but are physically separated, allow the exchange of soluble factors without direct contact. This setup is advantageous for studying paracrine effects and may avoid inhibitory effects from direct cell interactions, though the absence of direct contact may weaken osteogenic signaling compared to direct coculture [44]. From a practical perspective, our data suggest that CM is particularly suitable when the goal is to enhance osteogenic differentiation while limiting extensive cell expansion, for example, in preseeded scaffolds where space is constrained. Direct coculture may be preferable when strong bidirectional cell–cell interactions are desired and early proliferation is beneficial, whereas indirect coculture maximizes paracrine signaling and mineralized matrix formation without direct contact. In contrast, ODM remains a robust positive control but may not fully recapitulate the complex paracrine effects observed with CM‐based or coculture approaches.

## 5. Conclusion

In summary, our findings indicate that the CM and coculture systems (particularly the indirect setup) are highly effective strategies to promote osteoblastic differentiation in DPSCs and Saos‐2, albeit with distinct effects on proliferation: CM tends to slow down cell growth while enhancing differentiation, whereas coculture simultaneously supports cell expansion and the acquisition of an osteogenic phenotype. Direct and indirect coculture methods, alongside CM treatment, offer promising approaches for tissue engineering and regenerative medicine, particularly in bone and dental tissue repair. The optimal choice depends on the specific application requirements, including the necessity for cell‐to‐cell contact and the desired osteoinductive signaling profile.

## Author Contributions

**Lina M. Escobar**: conceptualization, methodology, formal analysis, writing – original draft, writing – review and editing, project administration. **Zita Bendahan**: methodology, investigation, data curation. **Wendy Lugo**: validation, investigation, writing – review and editing. **Eliana Calderón and Luz Eliana Ramirez:** acquisition of data, analysis, interpretation. **Sandra Castaño:** concept and design of study, drafting, revision. **Jaime E. Castellanos**: conceptualization, supervision, writing – review and editing. **Ma Clara Gonzalez-Carrera**: interpretation, drafting.

## Funding

This study was supported by grants from the Universidad El Bosque (Grant PCI 2022‐11044).

## Disclosure

All authors have read and approved the final version of the manuscript. Lina M. Escobar: corresponding author, had full access to all of the data in this study and takes complete responsibility for the integrity of the data and the accuracy of the data analysis.

## Conflicts of Interest

The authors declare no conflicts of interest.

## Data Availability

The data that support the findings of this study are available from the corresponding author upon reasonable request.
